# Incidence and determinants of nosocomial infection among hospital admitted adult chronic disease patients in University of Gondar Comprehensive Specialized Hospital, North–West Ethiopia, 2016–2020

**DOI:** 10.3389/fpubh.2023.1087407

**Published:** 2023-02-24

**Authors:** Zewdu Wasie Taye, Yaregal Animut Abebil, Temesgen Yihunie Akalu, Getahun Mengistu Tessema, Eden Bishaw Taye

**Affiliations:** ^1^Health Field Officer at the International Committee of the Red Cross, Gondar, Ethiopia; ^2^Department of Epidemiology and Biostatistics, Institute of Public Health, College of Medicine and Health Sciences, University of Gondar, Gondar, Ethiopia; ^3^Department of Internal Medicine, University of Gondar Comprehensive Specialized Hospital, North–West Ethiopia, Gondar, Ethiopia; ^4^Department of Clinical Midwifery, School of Midwifery, College of Medicine and Health Sciences, University of Gondar, Gondar, Ethiopia

**Keywords:** chronic illness, nosocomial infection, incidence rate, determinants, University of Gondar Comprehensive Specialized Hospital

## Abstract

**Background:**

Nosocomial infections are major public health problem which affects more than 100 million patients each year globally. This leads to prolonged hospital stays, a high mortality rate, and a vast financial burden to the healthcare system as well as the patients. This study aimed to find out the incidence of nosocomial infections and determinant factors among admitted adult chronic illness patients at the University of Gondar Comprehensive Specialized Hospital, Northwest Ethiopia.

**Methods:**

An institutional-based retrospective follow-up study design was employed among 597 respondents. The secondary data was collected from April 15 to May 15, 2021. A computer-generated random sampling technique was used to select a total of 599 patients using Open-epi software. Structured checklists were used to collect data. For data entry and analysis Epi-Data version 4.6 and STATA 16 were used respectively. To identify statistically significant variables Cox-regressions (univariable and multivariable) were performed. To declare statistically significant variables based on *p* < 0.05 in the multivariable Cox-regression model, adjusted hazard ratio with 95% CI was used.

**Results:**

A total of 597(99.6%) adult chronic illness patients were included in the study. Of these, 53 (8.88%) participants developed nosocomial infections and the incidence rate of nosocomial infection was 6.6 per 1,000 person-days observation. In this study, not taking antibiotics (AHR = 2.74, 95% CI: 1.49, 5.04), using mechanical ventilation (AHR = 2.67, 95% CI: 1.36, 5.26), being on urinary catheter (AHR = 4.62, 95% CI: 2.22, 9.65), being on intravenous catheter (AHR = 3.42, 95% CI: 1.22, 9.61) and length of hospital stay >20 days (AHR = 2.66, 95% CI: 1.43, 4.94) were significantly associated with nosocomial infections.

**Conclusions:**

The findings have indicated that the incidence of nosocomial infection was low. No taking antibiotics, intravenous insertion, mechanical ventilation, length of hospital stay, and urinary catheterization were the predictors for the development of nosocomial infection. Therefore, we recommend that the healthcare providers need to give emphasis on infection prevention and control in the institution on these factors that have a significant effect on nosocomial infection.

## Introduction

Nosocomial infections (NIs) or healthcare associated infections are infections developed during the process of getting health care which occures 48 h after hospital admission without any evidence that the infection was existing at the time of hospital admission (1–3). The types of health care associated infections are catagorised as Catheter-associated urinary tract infections (CAUTI), Central line-associated bloodstream infections (CLABSI), Ventilator-associated pneumonia (VAP), Surgical site infections (SSI), and hospital acquired C.difficile infectons, (HO-CDI). Soft tissue infection, upper respiratory tract infection, central nervous tract infection, and reproductive tract infections may occur as health care associated infections (3).

Globally, nosocomial infections affect more than 100 million patients each year (4). In developed countries, the burden of NIs shows that more than four million patients are affected every year. It mostly affects high-risk admitted patients (3, 5). In a national and multi-center study by the World Health Organization in 2011, reported that the prevalence of nosocomial infections in hospital admitted patients varied from 3.5 to 12% in developed countries of Australia, France, Canada, Germany, Belgium, Finland, and others. A meta-analysis study showed that infections that occur associated with healthcare were 7.6 episodes per 100 patients (4). However, in developing countries, the prevalence of NIs varied from 3 to 15% (6).

In low- and middle-income countries, incidence of NI is as high as 35.2% which ranges from 4.4 up to 88.9%, which affects high-risk populations or those who are admitted to the hospitals. This remains higher in many folds of low and middle-income countries than high income ones (2). In developing countries few studies have focused on NIs, especially in Sub-Saharan Africa. A recent meta-analysis study from 220 studies conducted in developing countries (only 14 studies were from Africa) showed that the incidence rates of nosocomial infections was 7.4 infections per 100 patients (7).

In sub-Saharan Africa the incidence of nosocomial infections ranges from 2 to 49% (8). In Ethiopia, the incidence and prevalence of nosocomial infection are 35.8% (1) and 16.96% respectively (9). In Jimma University hospital wards, the incidence rate of nosocomial infection was 28.15% per 1000 patient days while the overall prevalence was 19.41% (10).

Nosocomial infection has becomes a huge healthcare problem which causes great economic and production loss in the community (11). The impact of NI implies long-term disability and prolonged hospital stay for individuals. In the community and government, it leads to a massive additional financial burden for health systems, increased resistance of microorganisms to antimicrobials, high costs for patients and their families, and increased deaths of patiants. In developed nations, NIs cause 16 million additional days of hospital stay, € 7 billion financial loss, and 37 000 attributable deaths every year (4). But, no clearly defined figures/numbers of nosocomial infections in developing nations due to limited data and low quality of data (12).

In the 21st century, nosocomial infections are more alarming due to the reasons of; hospitals serving a large number of people who are sick and have lower immunity, invasive medical procedures, poor hygiene practice, and routine use of antimicrobial agents (13). Nosocomial infections are still a major public health problem, because of antimicrobial resistance to pathogens (14).

Africa has a less effective infection control program due to: lack of personnel, lack of awareness, poor water supply, poor laboratory backup, ineffective antibiotic policies with the emergence of multiple antibiotic-resistant microbes, poor funding, and non-adherence to safe practices by health workers (15).

Even though there are declarations and interventions for the sustainable development goal of Ethiopia on nosocomial infections through an integration, monitoring, programmed prevention mechanisms, the preventive measures are still low. These preventive mechanisms include:—limiting transmission of organisms between patients, indirect patient care, protecting patients with appropriate use of prophylactic antimicrobials, controlling environmental risks for infection, vaccinations, and nutrition. Other preventive mechanisms also include:- limiting the risk of endogenous infections by minimizing invasive procedures and promoting optimal antimicrobial use, enhancing staff patient care practices, prevention of infection in staff members, and continuing staff education (16). In developing countries, especially in Africa, incidence of nosocomial infection is still high (1). These are due to negligence in useing infection prevention guidelines, financial problems to facilitate the infrastructures including medical equipment, wardrooms as well as personal protective equipment (15).

In Ethiopia, there are little evidences on the incidence and determinants of nosocomial infections. In addition, there are limited studies in the areas regarding incidence and determinants of nosocomial infections among hospital admitted adult chronic illness patients and with this method of analysis.

Therefore, this study aims to determine the incidence and determinants of nosocomial infections in the University of Gondar Comprehensive Specialized Hospital, Northwest Ethiopia.

## Methods

### Study area

The study was conducted at the University of Gondar Comprehensive Specialized Hospital, Amhara regional state, Northwest Ethiopia. The University of Gondar Comprehensive Specialized Hospital is found in Gondar city, which is found 738 kilometers (km) from the Northwest of Addis Ababa, the capital city of Ethiopia. It gives services to more than seven million persons in the catchment area (17). The hospital has specializations in pediatrics, internal medicine, surgery, gynecology, and other health related specializations and it serves more than five million people in inpatient and outpatient departments (18). The health service units include maternity clinics, outpatient clinics, adult in-patients, emergency wards, community clinics, pediatrics in-patients, radiology, dermatology, pathology, ophthalmology, dentistry, pharmacy and medical laboratory (19). The hospital has about 700 beds in 27 wards for inpatient, emergency, and outpatient departments (17). It is staffed by about 1,040 health care professionals (20).

### Study design and period

A retrospective follow-up study was conducted among adult chronic illness patients admitted in the medical wards from January 01, 2016 to December 31, 2020.

### Participants

All adult chronic illness patients who were admitted to the University of Gondar Comprehensive Specialized Hospital were the source populations, while all adult chronic illness patients who were admitted at the University of Gondar Comprehensive Specialized Hospital between 2016–2020 were the study populations.

### Sample size calculation and sampling procedures

The sample size was calculated by using the formula for survival analysis by considering the following statistical assumptions:

*N* = required sample size, ${\text{Z}}_{\frac{\alpha }{2}}$ = 1.96, the corresponding Z-score for the 95% CI, m = events of nosocomial infection = 448, P_m_ = Proportion events of nosocomial infection = 0.86 taken from the previous study conducted in Debre-Markos Referral Hospital, North-west Ethiopia (21), HR = Hazard ratio = 1.32, *Z*_β_ = the critical value of the standard normal distributed variable at 20% of β, which is the probability of type two error (0.8416),


$$\begin{array}{l} m=4\frac{\left(Z\beta +\frac{Z\alpha }{2}\right)2}{\left(\theta \right)2},and\;n\;=\frac{m}{P\left(m\right)} \end{array}$$

By considering non-response rate 15% (22), the calculated maximum sample size was (521^*^0.15) = 78; then 521 + 78 = 599.

A computer-generated random sampling technique was used to select a total of 599 patients using Open-epi software.

### Inclusion and exclusion

All admitted adult chronic illness patients between January 01, 2016 to December 31, 2020 and who have no new signs and symptoms of infection before 48 h of admission were included. Adult chronic illness patients who had missing admission dates and discharge dates were excluded.

### Operational definitions

#### Nosocomial infections

Infections acquired in the hospital by a patient during a hospital stay.

#### Incidence of nosocomial infection

The number of NIs occurring among admitted adult chronic illness patients from 2016 to 2020.

#### Chronic illness

An illness of patients who have at least one of the following (hypertensive, or type-I and type-II DM or cardiac, or COPD, or asthma, or renal, or liver disease).

#### Event

Development of nosocomial infections following admission until discharge.

#### Censored

Patients have not developed nosocomial infections from admission to the time of discharge or who were lost before developing any nosocomial infection.

#### Length of hospital stay

The time of a patient that stays admitted to the hospital.

#### Invasive medical devices

Medical devices like urinary catheter or IV-catheter or chest tube or NG-tube or mechanical/nasal ventilator.

### Study variables

The outcome variable of this study was time to nosocomial infections (event, censored), while others like socio-demographic, behavioral factors, clinical and healthcare-related factors were the explanatory variables.

### Data collection tools and procedures

Data extraction formats were used to collect the data that was prepared in English. The checklist/formats were adapted from different literatures. Data were collected by BSc nurses and followed by the supervisor who managed the overall data collection process. A one-day training was given to the supervisor and data collectors about data collection tools, the purpose of the study, data collection techniques, collection of the data, and ethical issues. The supervisor assessed the completeness and consistency of data daily. All detailed data like socio-demographic, behavioral, and clinical, and health-related variables were reviewed from the charts of hospital-admitted adult chronic illness patients.

### Statistical analysis

The data entry was performed using Epi-Data version 4.6 and then exported into STATA 16 for analysis. Descriptive statistics like mean, median, frequency, and percentage were used to present variables using texts, tables, and graphs. A *p*-value of ≤ 0.05 was used as statistically significant. To identify the determinants of nosocomial infection, Cox proportional hazard model was used. To identify statistically significant variables, Cox regression (Bivariable and multivariable) was performed using a *p* value ≤ 0.2 in the univariable Cox regression analysis to identify candidate variables for multivariable Cox regression. To declare statistically significant variables, an adjusted hazard ratio with 95% CI was used, based on *p* value < 0.05 in the multivariable Cox regression analysis. The goodness of fit of the model was tested by using the Cox-Snell residuals together with Nelson Aalen's cumulative hazard function.

### Quality assurance mechanisms

To assure the quality of the data, the tool was prepared first by language experts in English. Data collectors and the supervisor were trained in the data collection process for 1 day. A pretest was conducted from 5% of the total sample size in the hospital which was selected for actual data collection. Appropriate modifications were made to the tool accordingly. Data collection was closely monitored by investigators and the supervisor. Moreover, the data quality was assured by using statistical parameters to assess the consistency and completeness of the data.

### Ethics approval and consent to participate

Ethical clearance was obtained from the Institutional Review Board (IRB) of the University of Gondar on behalf of the ethical review committee of the Department of Epidemiology and Biostatistics. A formal letter of cooperation from the University of Gondar was delivered to the hospital and permission was obtained from the hospital administration. Wavering was taken from the referral hospital after detailed information was provided about the objective of the study and the data collection was started and the study was started after complete consent is obtained.

## Results

### Respondents' sociodemographic characteristics

Of the overall sample required (*N* = 599), 597 participants were included in the study, giving a response rate of 99.6%. The mean age of the participants was 51.7 years with SD ± 18.6. Among all respondents, of all, 305 (51.09%) were males and 418 (70.02%) were married. Regarding educational status, 344 (57.62%) were unable to read and write. Moreover, 127 (21.27%) were housewives by occupation (Table 1).

**Table 1:** Socio-demographic and baseline behavioral characteristics among adult chronic illness patients admitted at the University of Gondar Comprehensive Specialized Hospital, Northwest Ethiopia, 2016–2020 (N = 597).

**Variables**	**Responses**	**Frequency (%)**
**Age (years)**		
	18–30	116 (19.43)
	31–45	115 (19.26)
	>45	366 (61.31)
**Sex**		
	Male	305 (51.09)
	Female	292 (48.91)
**Marital status**		
	Married	418 (70.02)
	Unmarried	97 (16.25)
	Divorced	21 (3.52)
	Widowed	61 (10.22)
**Educational status**		
	Unable to read and write	344 (57.62)
	Able to read and write	253 (42.38)
**Occupation**		
	Housewife	127 (21.27)
	Merchant	51 (8.54)
	Farmer	135 (22.69)
	Daily laborer	14 (2.35)
	Government employee	31 (5.19)
	Student	28 (4.69)
	Unemployed	8 (1.34)
	Unknown	203 (34)
**Residence**		
	Urban	255 (42.71)
	Rural	342 (57.29)
**History of alcohol drinking**		
	Yes	48 (8.04)
	No	549 (91.96)
**History of cigarette smoking**		
	Yes	5 (0.84 )
	No	592 (99.16)
**History of chat chewing**		
	Yes	8 (1.34)
	No	589( 98.66)

### Clinical and health-related characteristics of chronic illness patients

Of all, 26 (4.36%) and 82 (13.74%) had type I and type II diabetes mellitus, respectively. Of all, 236 (39.53%) had cardiac disease. Fifty-five (9.21%) of the study subjects had chronic renal disease. More than a quarter of the study participants, 174 (29.15%) had hypertension. Among the total participants, 47(7.85%), 32(5.36%), and 28 (4.67%), were admitted to the hospital due to stroke, shock, and severe pneumonia respectively. Among the total admitted patients, 423 (70.85%) had an intravenous catheter during admission and 524 (87.48%) received antimicrobial medication. Concerning invasive medical devices, 49 (8.21%) were applied to intervention. From the total, 53 nosocomial infection patients, 3 (5.66%), 48 (90.57%), and 2 (3.77%) were died, improved, and lost to follow up respectively (Table 2).

**Table 2:** Clinical factors among adult chronic illness patients admitted at the University of Gondar Comprehensive Specialized Hospital, Northwest Ethiopia, 2016–2020 (*N* = 597).

**Variables**	**Response**	**Number of patients developed NIs (%)**	**Number of censored (%)**	**Frequency (%)**
**Type I DM**				
	Yes	0	27 (4.52)	27 (4.52)
	No	53 (8.88)	517 (86.6)	570 (95.48)
**Type II DM**				
	Yes	7 (1.17)	75 (12.52)	82 (13.74)
	No	45 (7.54)	470 (78.73)	515 (86.26)
**Cardiac disease**				
	Yes	25 (4.19)	211 (35.34)	236 (39.53)
	No	27 (4.52)	334 (55.94)	361 (60.47)
**Chronic Renal disease**				
	Yes	6 (1.0)	49 (8.2)	55 (9.21)
	No	46 (7.7)	496 (83.08)	542 (90.79)
**Chronic liver disease**				
	Yes	13 (2.18)	74 (12.4)	87 (14.57)
	No	39 (6.53)	471 (78.89)	510 (85.43 )
**Asthma**				
	Yes	2 (0.33)	20 (3.51)	22 (3.69)
	No	51 (8.54)	524 (87.65)	575 (96.31)
**COPD**				
	Yes	0	42 (7.04)	42 (7.04)
	No	53 (8.88)	502 (84.09)	555 (92.96)
**Hypertension**				
	Yes	11 (1.84)	163 (27.3)	174 (29.15)
	No	41 (6.87)	382 (63.99)	423 (70.85)
**History of previous admission**				
	Yes	21 (3.51)	152 (25.46)	173 (28.98)
	No	32 (5.36)	392 (65.66)	424 (71.02)
**Conditions of index hospital admission**				
**Stroke**				
	Yes			47 (7.87)
	No			550 (92.13)
**Shock**				
	Yes			32 (5.36)
	No			565 (94.64)
**Asthma**				
	Yes			6 (1.01)
	No			593 (98.99)
**Hypertension**				
	Yes			7 (1.17)
	No			590 (98.83)
**Cardiac disease**				
	Yes			26 (4.36)
	No			571 (95.64)
**DM foot ulcer**				
	Yes			44 (7.37)
	No			553 (92.63)
**Intervention at the time of admission**				
**Urinary catheter insertion**				
	Yes			19 (3.18)
	No			578 (96.82)
**Intravenous catheter insertion**				
	Yes			423 (70.85)
	No			174 (29.15)
**Surgery after admission**				
	Yes			2 (0.34)
	No			595 (99.66)
**Mechanical ventilation**				
	Yes			45 (7.54)
	No			552 (92.46)
**Patient received antimicrobial medication**				
	Yes			524 (87.48)
	No			73 (12.52)
**Length of hospital stay in days**				
	3-20			488 (81.74)
	>20			109 (18.26)
**Outcome of the admitted patients**				
	Died			14 (2.35)
	Improved			581 (97.32)
	Unknown			2 (0.34)

### Incidence of nosocomial infection

The overall incidence rate of nosocomial infection was 6.6 cases (95% CI: 5.1, 8.7) per 1,000 person-days observation with the prevalence of 8.88%. Study subjects were followed for a total of 7,984 person-days observation with a median time of 10 days (IQR = 7–16 days).

### Types of nosocomial infection in respect to chronic illness

**Figure 1:**
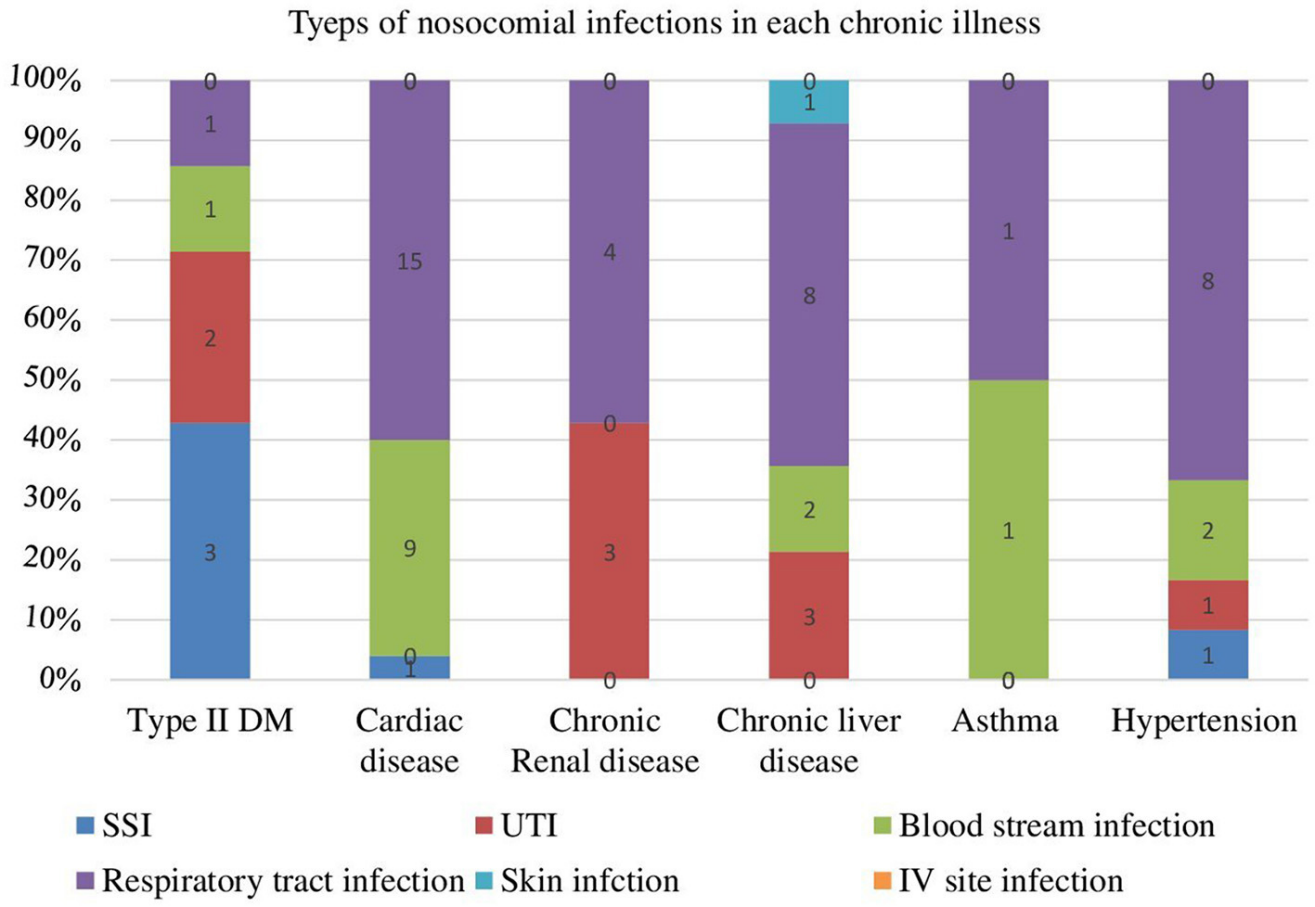
Types of nosocomial infection among chronic illness patients at University of Gondar Comprehensive Specialized Hospital Northwest Ethiopia from 2016–2020 (*N* = 53).

In this study the most common types of nosocomial infection were respiratory tract infection or hospital acquired pneumonia 30 (56.6%) and blood stream infection 13 (24.53%). An estimated 13.21, 5.66, and 1.88% of the participants were developed urinary tract infection, surgical site infection and skin infection respectively (Figure 1).

### Factors associated with the development of nosocomial infection

From the multi-variable Cox-regression analysis, 5 predictor variables: length of hospital stay (>20 days), urinary catheter insertion, intravenous catheter insertion, mechanical ventilation, and no use of antibiotics during admission were found associated with the occurrence of nosocomial infection at 95% CI with *P*-value ≤ 0.05.

In our study, the hazard of developing NIs among hospital admitted adult chronic illness patients who had a urinary catheter was 4.62 times **(**AHR = 4.62, 95% CI: 2.22, 9.65) higher compared to non-urinary catheterized patients. The hazard of developing NIs among hospital admitted adult chronic illness patients who have an intravenous catheter was 3.42 times (AHR = 3.42, 95% CI: 1.22, 9.61) higher compared to the patients with no intravenous catheter. The hazard of developing NIs among hospital admitted adult chronic illness patients who have mechanical ventilation during admission was 2.67 times (AHR = 2.67, 95% CI: 1.36, 5.26) higher than the patients with no mechanical ventilation. The hazard of developing NIs among hospital admitted adult chronic illness patients who did not take antibiotics during admission was 2.74 times (AHR = 2.74, 95% CI: 1.49, 5.04) higher compared to patients who took. The hazard of developing NIs among hospital admitted adult chronic illness patients who had >20 days of hospital stay was 2.66 times (AHR = 2.66, 95% CI: 1.43, 4.94) higher compared to patients who stayed < 20+ days of admission in the hospital (Table 3).

**Table 3:** Factors affecting the occurrence of nosocomial infection among adult chronic illness patients admitted at University of Gondar Comprehensive Specialized Hospital, Northwest Ethiopia, 2016–2020 (*N* = 597).

**Variables**	**NI**		**CHR(CI)**	**AHR**	**95% CI**

	**Yes**	**No**			
**Urinary catheter**					
Yes	9	10	6.95 (3.37, 14.32)^**^	4.62	(2.22, 9.65)^*^
No	44	534	1	1	
**IV Catheter**					
Yes	49	374	4.56 (1.64, 12.65)^*^	3.422	(1.22, 9.61)^*^
No	4	170	1	1	
**Age**					
18–30	19	97	1	1	
31–45	8	107	0.555 (0.241, 1.276)	0.568	(0.22, 1.50)
>45	340	26	0.524 (0.288, 0.952)	0.517	(0.25, 1.08)
**Occupation**					
Housewife	7	120	1	1	
Merchant	3	48	1.158 (0.297, 4.519)	1.756	(0.44, 7.07)
Farmer	12	123	1.91 (0.748, 4.882)	2.37	(0.91, 6.19)
Daily laborer	3	11	3.889 (0.997, 15.162)	2.54	(0.57, 11.44)
Govn't employed	2	29	1.08 (0.223, 5.238)	1.57	(0.30, 8.09)
Student	3	25	1.80 (0.463, 7.01)	0.95	(0.21, 4.39)
Unemployed	2	6	7.584 (1.5547, 7.18)	4.44	(0.95, 37.86)
Unknown	21	182	2.39 (1.01, 5.68)	2.68	(0.99, 6.57)
**Chronic liver disease**					
Yes	13	74	2.043 (1.089, 3.835)	2.17	(0.89, 4.30)
No	40	470	1	1	
**Mechanical ventilation**					
Yes	11	34	3.78 (1.94, 7.38)^**^	2.67	(1.36, 5.26)^**^
No	42	510	1	1	
**Antibiotics**					
Yes	38	481	1	1	
No	15	63	3.053(1.669, 5.587)^**^	2.742	(1.493, 5.035)^**^
**Length of hospital stay in days**					
3–20	21	467	1	1	
>20	32	77	2.974(1.605, 5.511)^**^	2.661	(1.433, 4.942)^**^

^*^p-value < 0.05, ^**^p-value ≤ 0.001.

## Discussion

This study assessed the incidence of nosocomial infection and examined the socio-demographic, behavioral, and clinical determinants of NIs among adult hospital admitted chronic illness patients at the University of Gondar comprehensive and specialized hospital. In this study, the overall incidence rate of NIs was 6.6 (95% CI: 5.1, 8.7) per 1,000 person-days of observation. This finding is in-line with the study conducted in Sab-Saharan Africa (8) and Southeast Asia, Taiwan (23).

This finding was higher than studies conducted in low-income countries like Nepal (24) and Gabon (25). Moreover, the finding was higher than studies conducted in high-income countries of Southeast Asia (South Korea and Japan) (23) and Trinidad-Tobago (26). This higher incidence of NIs also could be due to poor infrastructure and maybe poor adherence to aseptic measures that facilitate the transmission and occurrence of nosocomial infections.

On the other hand, this finding was lower than a study conducted in Ethiopia (10), a review done in Africa (27), Kenya (28), Cameron (29), and Northern India (30). The difference could be due to study time, which currently is high laboratory access and different infection prevention infrastructures that have a high quality of detection and prevention of Nis, respectively. The level of development of health system affects the occurrence of NIs due to the presence or absence of equipments, as well as NIs control measurements (31). The other reason may be due to differences in study participants; in those studies the participants were in different wards, such as ICU, emergency wards, and surgical wards, which may be a higher risk of developing NIs (27).

This study revealed that patients who had urinary catheters were 4.62 times at higher hazard of developing NIs compared with non-catheterized chronic patients. This result is consistent with other studies done in Morocco (32), Egypt (31, 33), Poland (34), China (35), Pune India (36), and Northern India (30). This may be due to microorganisms causing NIs deriving from the patient's perineal flora or the hands of healthcare personnel during catheter insertion or manipulation of the collection system by direct inoculation, or by ascending organisms from the perineum by capillary action in the thin mucous film contiguous to the external catheter surface. Reflux of microorganisms gaining access to the catheter lumen causes intraluminal contamination from contamination of urine in the collection bag or the failure of closed drainage (37).

In this study, patients who had intravenous catheters were 3.42 times at higher hazard of developing NIs compared with non-catheterized chronic patients. The study was supported by different studies conducted in Morocco (32), Egypt (31, 33), Poland (34), Switzerland (38), and Northern India (30). This may be due to bacterial dissemination along with the tube into the patient's body. An intravenous catheter may become blocked, leak fluid into the skin, and cause infection. Skin colonization is a significant source of central venous catheter colonization and infection (39).

Patients who had mechanical ventilation during admission were 2.67 times at higher hazard of developing NIs compared with chronic patients without mechanical ventilation. This was supported by a study done in Adama (40), Jimma (10), Egypt (33), Brazil (41), China (35), Pune India (36), and Northern India (30). Patients who receive mechanical ventilation have a markedly increased risk of developing NIs. Both nasotracheal and orotracheal tubes bypass natural host defenses which permit leakage of bacteria and secretions around the cuff into the trachea that leads to damage the ciliated epithelium of the trachea and decrease bacterial clearance (42). This is due to the colonization of pathogens into the oropharynx and its contiguous structures, like dental plaque, sinuses, gastric reservoir and trachea, and aspiration of the contaminated secretions into the lower airway causing NIs (43).

In this study, patients who did not take antibiotics during admission were 2.74 times at higher hazard of developing NIs compared with chronic patients who took. The finding was supported by the study conducted in Italy (44). The reason may be due to antibiotic prophylaxis being used to prevent infections by preventing and eradicating carriage of aerobic, pathogenic micro-organisms from the oropharynx, stomach, and gut (44). On the contrary, a study which was conducted in Bahir Dar (45) and the finding observed in Morocco (32) stated that patients who received antibiotics prophylaxis were more at risk for the occurrence of NIs than those not received prophylaxis. This may be due to the entrance of drug-resistant bacteria or pathogens. Broad-spectrum containing antimicrobials prescribed to patients increased the risk of hospital-acquired drug-resistant infections (46).

Lastly, patients who stayed >20 days admitted in the hospital were 2.66 times at higher hazard of developing NIs compared with those chronic patients who stayed < 20 days. The finding was supported by other studies done in Africa (6), Morocco (32), Tunisia (47), Scotland (48), Pune India (36), Egypt (31), Bahir Dar (45), Jimma (10), Tanzania (49), and Wolaita-Sodo University teaching hospital (50). This is due to the long duration of hospital stay makes patients to be exposed to environment pathogens which increases cross-contamination and increases the patient's susceptibility to hospital-acquired infections. In addition, an increase in duration of stay among those who had underlying morbidity and those who required invasive procudgers (51).

### Limitations of the study

To conduct the study secondary data was used for this reason important variables like nursing care given to the patients, patient-to-bed ratio, hygiene, and sanitation couldn't be assessed.

In this study, isolating organisms laboratory culture was not used which is a guide to confirm the results of Nis. This could have affected our results.

## Conclusions

The incidence rate of nosocomial infection among chronic illness patients in the University of Gondar Comprehensive Specialized Hospital was low. Patients who did not take antibiotics during admission, intravenous insertion, mechanical/nasal ventilation, length of hospital stay (>20 days), and urinary catheterization were significant predictors for the development of nosocomial infection among adult chronic illness patients.

## Data availability statement

The datasets presented in this article are not readily available because we generate the data in reasonable request. Requests to access the datasets should be directed to ZT, zewasie7@gmail.com.

## Ethics statement

The studies involving human participants were reviewed and approved by the Institutional Review Board (IBR) of the University of Gondar on behalf of the ethical review committee of the Department of Epidemiology and Biostatistics. The Ethics Committee waived the requirement of written informed consent for participation.

## Author contributions

ZT, YA, TA, and ET wrote the proposal, participated in data collection, analyzed the data, drafted the paper and prepared the manuscript, approved the proposal with few revisions, participated in data analysis, and revised subsequent drafts of the paper. GT wrote the proposal, participated in data collection, and approved the proposal with few revisions. All the authors read and approved the final manuscript.
